# A Novel LncRNA, *MuLnc1*, Associated With Environmental Stress in Mulberry (*Morus multicaulis*)

**DOI:** 10.3389/fpls.2018.00669

**Published:** 2018-05-29

**Authors:** Ying-Ping Gai, Shuo-Shuo Yuan, Ya-Nan Zhao, Huai-Ning Zhao, Hua-Liang Zhang, Xian-Ling Ji

**Affiliations:** ^1^State Key Laboratory of Crop Biology, Shandong Agricultural University, Tai’an, China; ^2^College of Forestry, Shandong Agricultural University, Tai’an, China

**Keywords:** mulberry (*Morus* spp), lncRNAs, miR3954, siRNA, calmodulin-like protein gene *CML27*, environmental stress

## Abstract

Environmental stresses are major constraints that limit the leaf productivity and quality of mulberry. LncRNAs have emerged as important regulators in response to biotic and abiotic stresses in plants. However, the functions and mechanisms of most lncRNAs remain largely unknown. A novel lncRNA designated as *MuLnc1* was found to be cleaved by mul-miR3954 and produce secondary siRNAs in a 21 nt phase in mulberry. It was demonstrated that one of the siRNAs produced, si161579, can silence the expression of the calmodulin-like protein gene *CML27* of mulberry (*MuCML27*). When *MuCML27* was heterologously expressed in Arabidopsis, the transgenic plants exhibited enhanced resistance to *Botrytis cinerea* and *Pseudomonas syringae* pv tomato DC3000. In addition, the transgenic *MuCML27-*overexpressing Arabidopsis plants are more tolerant to salt and drought stresses. Furthermore, the network of mul-miR3954-*MuLnc1*-siRNAs-mRNAs was modeled to elucidate the interaction between lncRNAs and sRNAs with mRNAs. All of these, taken together, suggest that *MuLnc1* was associated with environmental stress in mulberry and may be considered as a potential genetic improvement target gene of mulberry. The information provided may shed light on the complicated gene expression regulatory mechanisms in mulberry stress responses.

## Introduction

Mulberry (*Morus* spp), an important food plant of the silkworms (*Bombyx mori*), is widely cultivated throughout the world in subtropical and temperate regions and is subjected to various environmental stresses during its entire life cycle (Ji et al., 2010). These stresses represent the leading constraints to leaf quality and productivity of mulberry, and feeding these stressed leaves affects larval development and cocoon characters of silkworm (Philip et al., 1994). Thus, it is fundamental to develop mulberry varieties with increased tolerance to environmental stresses to improve mulberry characteristics. Plant has developed complex signal transduction pathways to adapt to environmental stresses, and it is important to elucidate the expression networks of the genes involved in the signal transduction pathways to develop strategies to enhance the crop tolerance to environmental stresses (Xiong et al., 2002). However, the genetic control of tolerance to biotic and abiotic stresses is very complex, and the plant’s response to these stresses is accompanied by the activation of many genes associated with the perception and transduction of stress signals and by modulation of expression of defense-related genes (Atkinson and Urwin, 2012).

Previous research on molecular mechanisms underlying plant stress tolerance is largely focused on the functions of protein-coding genes (Wang J. et al., 2015). Recently, however, a large number of non-coding RNAs (ncRNAs) have been identified and found to play important roles in various biological processes including plant responses to environmental stresses (Ghildiyal and Zamore, 2009; Zhu and Wang, 2012; Joshi et al., 2016; Zhao et al., 2016). The ncRNAs comprise a class of RNAs such as miRNAs, siRNAs, ta-siRNAs and long non-coding RNAs (lncRNAs) (Axtell, 2013; Hirose et al., 2014; Bazin et al., 2017). Plant miRNAs are a class of small ncRNAs (20–24 nucleotides) which negatively regulate their targets via either mRNA degradation or translational repression (Pasquinelli, 2012). Besides guiding cleavage of target mRNA, it has been demonstrated that some miRNA-mediated cleavages of transcripts are capable of triggering the production of secondary siRNAs called trans-acting siRNAs (ta-siRNAs). ta-siRNAs can regulate the expression of their target genes to which they have limited sequence similarity in a way similar to miRNA (Yoshikawa et al., 2005; Manavella et al., 2012; Zhao et al., 2015). In contrast to miRNAs and siRNAs, lncRNAs are mRNA-like transcripts longer than 200 nucleotides that do not encode proteins (Quinn and Chang, 2016). In the last few years, lncRNAs have been considered as important regulators implicated in modulating transcription and as well as regulation of gene expression at post-transcriptional level, such as modulation of RNA stability and translation (Ponting et al., 2009; Wang and Chang, 2011; Heo et al., 2013; Wang T. et al., 2015; Chen et al., 2016). There were evidences showed that some lncRNAs serve as precursors of miRNAs and siRNAs which can perform their regulatory functions (Hirsch et al., 2006; Ma et al., 2014). Moreover, some lncRNAs harbor potential miRNA regulatory elements and are potentially targets of miRNAs or act as endogenous target mimics for miRNAs and participate in the miRNA regulatory network (Jalali et al., 2013; Wu et al., 2013; Wang J. et al., 2015). Therefore, ncRNAs can act as regulatory molecules involved in diverse biological processes and responses to biotic or abiotic stresses in plants (Mercer et al., 2009; Zhang et al., 2014; Wang T. et al., 2015; Chung et al., 2016; Joshi et al., 2016), and there are well-orchestrated regulatory interaction networks between various ncRNAs (Nacher and Araki, 2010; Collins, 2011; Jalali et al., 2013). However, in most cases, the interplay between the regulatory pathways of different ncRNAs remains largely unknown. As far as we know, there was only one report about the lncRNAs in mulberry so far (Song et al., 2016). Therefore, identification and characterization of stress-responsive ncRNAs and defining their regulatory networks will improve our understanding of the mechanisms underlying mulberry responses to environmental stresses.

In this study, based on the ncRNAs and transcriptome information attained, we report a novel lncRNA, *MuLnc1*, which was targeted and cleaved by mul-miR3954 and generated secondary siRNAs in a 21 nt phase in mulberry. One of the siRNAs produced, si161579, can direct the cleavage of the calmodulin-like protein gene *CML27* (*MuCML27*) transcript of mulberry. Furthermore, we modeled a network of interactions between miRNAs, lncRNAs, siRNAs and protein-coding mRNAs. It may provide a basis to further study of the complicated gene expression regulatory mechanisms in mulberry stress responses and to explore the genes and ncRNAs which have a great potential application value in mulberry biotechnology in future.

## Materials and Methods

### Plant Materials

One-year-old mulberry cutting seedlings of Husang 32 (*M. multicaulis*) were grown in a growth chamber at 26°C with 12 h of light and 90% humidity. *Arabidopsis thaliana* (Col-0) plants were incubated under standard greenhouse conditions (22°C, humidity 90% and 12 h of light).

### MiRNA Target Prediction and lncRNA Identification

The sixth leaves of five plants were sampled and mixed together, and the total RNA from the leaves sampled for transcriptome was isolated using TRIzol^®^ reagent (Invitrogen, Carlsbad, CA, United States). The cDNA library was constructed and sequenced according the method described by Xie et al. (2013). The sequences obtained by high-throughput sequencing were chosen to predict the mul-miR3954 targets using psRNATarget^1^. The predicted miRNA targets were validated using the degradome sequencing data of the leaves of Husang 32 with the software package, CleaveLand 3.0, as previously described (Addo-Quaye et al., 2008). The Coding Potential Calculator (CPC^2^), CNCl and Pfam were used to predict transcripts with coding potential, and the transcripts with CPC score<-1 and CNCl score <0 were discarded. The remaining transcripts with length >200 nt and open reading frames (ORFs) <80 bp were considered as lncRNAs. The secondary siRNAs were identified based on the sequences from small RNA library sequencing data of the leaves of Husang 32 according to the method described before (Gai et al., 2014).

### qRT-PCR Analysis

The TRIzol^®^ reagent (Invitrogen, Carlsbad, CA, United States) was used to extract total RNAs of the leaf samples, and the RNAs extracted were digested with DNase I before being used. qRT-PCR was performed on the Rotor-Gene 3000A instrument (Corbett Research, Sydney, Australia) with the SYBR Premix Ex TaqTM kit (Takara, Shanghai, China) for mRNA and the PrimeScriptTM miRNA qPCR Starter Kit Ver.2.0 (Takara, Shanghai, China) for miRNAs according to the manufacturer’s protocol. The actin and U6 genes were chosen as reference genes for normalization of mRNA and miRNA, respectively. The comparative cycle threshold (Ct) method (Livak and Schmittgen, 2001) was used to revaluate the relative gene expression levels. All samples were assayed in triplicate. The primes used for qRT-PCR are given in the **Supplementary Table S1**.

### RNA and Protein Gel Blotting

Total RNAs extracted were firstly separated on (1.2% w/v) formaldehyde denatured agarose gel and then were transferred onto nylon Hybond N membrane. The blots were hybridized with the probes labeled by digoxigenin (DIG) using the PCR DIG Probe Synthesis Kit (Roche, Germany). Prehybridization, hybridization with probe, washing, and detection steps were performed according to the protocol described by Liu et al. (2008). For the mul-miR3954, si165369, si47311, si161579 and si24218 northern blot analyses, sRNAs were extracted with miRCURY^TM^ RNA Isolation Kit (Takara, Shanghai, China). Specific digoxigenin-labeled RNA probes complementary to the mul-miR3954 and each siRNA were synthesized by Roche Ltd (Roche, Shanghai, China). Prehybridization, hybridization, washing, and detection were performed as previously described method (Yang et al., 2008). The probes used are given in the **Supplementary Table S2**.

The prepared proteins were mixed with 5 × SDS–PAGE sample buffer and heated for 3 min at 95°C and then were subjected to SDS/PAGE on 12% (w/v) polyacrylamide gels. After electrophoresis, proteins were transferred electrophoretically onto nitrocellulose membranes for protein gel blot analysis. Polyclonal anti-MuCML27 antibodies were generated by immunizing rabbits with purified MuCML27 protein, and the protein was detected by western blots using horseradish peroxidase (HRP)-conjugated polyclonal anti-MuCML27. Western blot analysis was conducted according to the procedure described by Poppenberger et al. (2011).

### Construction of si161579 Expression Vector

To study the function of si161579, the vector OE-si161579 over-expressing si161579 was constructed as described by Wang et al. (2010) with modifications. Firstly, a genomic fragment of the ath-miR165 backbone of 176 base pairs was amplified using primers miR165F and miR165R and cloned into pBI121. The mature ath-miR165-producing DNA region was substituted by si161579 using overlapping PCR with the combination of primers si161579 I, si161579 II, si161579 III, si161579 IV, miR165F and miR165R as described before (Schwab et al., 2006). The sequences of the primers used for the vector construction are listed in **Supplementary Table S3**.

### Production of Transgenic Plant Lines

The mature mul-miR3954-producing DNA region was cloned from mulberry DNA, and the RNA isolated from leaves of mulberry was used to synthesize cDNA which was used as template to clone the *MuCML27* and *MuLnc1* genes, respectively. Primers (**Supplementary Table S3**) used to amplify the genes were designed based on the gene sequences (**Supplementary Table S4**). The genes cloned were then ligated individually into the binary vector pBI121 under the control of the CaMV 35S promoter. Then the vector was introduced into *Agrobacterium tumefaciens* strain GV3101 and transformed into WT Arabidopsis plants by floral dipping. Transformants were selected and confirmed by qRT-PCR and northern blot. The T3 generations from independent ectopic expression transgenic lines were randomly chosen for further functional studies.

### Detection of Resistance Against Pathogens

The 4-week-old Arabidopsis plants were used for resistance analysis. Inoculation with *Pst* DC3000 was conducted by spraying the bacterial suspensions (10^5^ CFU mL^-1^) of *Pseudomonas syringae* pv tomato DC3000 (*Pst* DC3000) onto the rosette leaves. Bacterial growth within the leaf was monitored by a serial-dilution assay (Katagiri et al., 2002). Detached rosette leaves were inoculated with 2-mm-diameter mycelium plugs taken from the actively growing colonies of *Botrytis cinerea* grown on PDA medium and placed in a covered Petri dish to retain moisture and incubated at 22°C. Each treatment was conducted independently at least three times.

### Drought and Salt Stress

For drought treatments, Arabidopsis seedlings were grown in soil under regular watering regime for 4 weeks. Then the seedling plants were irrigated with 60 gL^-1^ PEG-6000 to simulate drought stress. For salt tolerance assay, 4-week-old Arabidopsis seedlings were irrigated with 1/8 concentrated MS salt solution supplemented with 250 mM NaCl. All the experiments were performed three times and three replicates for each treatment were used.

### Malondialdehyde and Proline Measurements

Fresh leaf material was boiled in 3% sulfosalicylic acid for 10 min with constant shaking. After cooling and filtering, the filtrate was used for proline content assay following the method described by Shan et al. (2007). For malondialdehyde (MDA) concentration measurements, fresh leaf material was homogenized using 5% (w/v) trichloroacetic acid and the homogenate was centrifuged at 12,000 × *g* for 15 min. The supernatant was mixed with an equal volume of thiobarbituric acid (0.5% in 20% [w/v] trichloroacetic acid) and then the mixture was boiled for 25 min at 100°C, followed by centrifugation for 5 min at 7,500 × *g*. The supernatant was used for MDA concentration measurement according to the procedure as described previously (Peever and Higgins, 1989). These experiments were repeated at least three times.

## Results

### *MuLnc1* Transcript as a Target of mul-miR3954 in Mulberry

**FIGURE 1:**
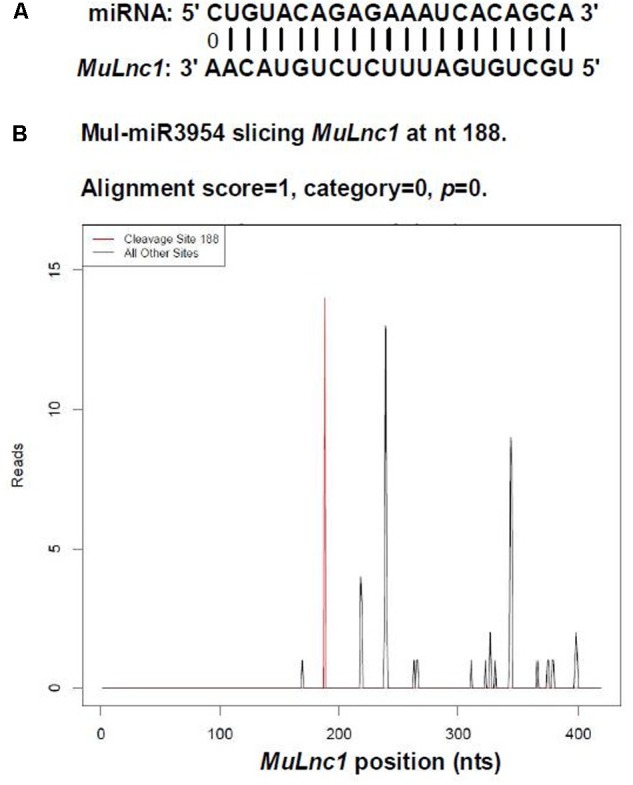
Identification of *MuLnc1* as a target of mul-miR3954 in mulberry. **(A)** Base-pairing interaction between mul-miR3954 and *MuLnc1*. **(B)** Validation of the *MuLnc1* degradation by degradome sequencing.

**FIGURE 2:**
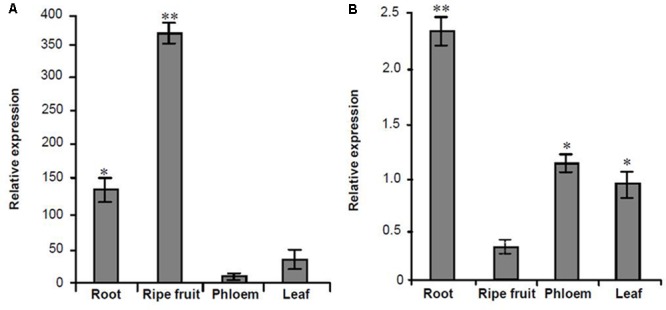
Quantitative analysis the expression levels of *MuLnc1* and mul-miR3954 in different tissues of mulberry using qRT-PCR. **(A)** *MuLnc1* abundance analysis by qRT-PCR. The relative gene expression was evaluated using comparative Ct method taking actin as the reference gene. **(B)** Mul-miR3954 abundance analysis by qRT-PCR. The relative abundance was evaluated using comparative Ct method taking U6 as the reference. Assays were performed three times, each time with three replicates. Values are given as the mean ± SD of three experiments in each group. Asterisks indicate significant difference based on Student’s *t*-test (^∗^*P* < 0.05, ^∗∗^*P* < 0.01).

Based on the transcriptome information attained, one lncRNA, *MuLnc1*, was identified. Interestingly, *MuLnc1* was predicted to be a target of mul-miR3954 (**Figure 1A**), and this was further experimentally verified by mRNA degradome sequencing (**Figure 1B**). To explore whether *MuLnc1* functioned as a target transcript of mul-miR3954, the correlation between the expression levels of *MuLnc1* and mul-miR3954 in mulberry tissues were examined by qRT-PCR. The results showed that *MuLnc1* was expressed at relatively high level in the ripe fruits, but its transcript level was very low in the roots, phloem and leaves (**Figure 2A**), whereas the expression level of mul-miR3954 was very low in the ripe fruits, but its transcript level was relatively high in the roots, phloem and leaves. This suggested that *MuLnc1* and mul-miR3954 expressions were highly tissue-specific, and there was a very good negative correlation between their expression levels (**Figure 2B**), and this is in accord with the function of miRNA in guiding target mRNA cleavage.

### Secondary siRNAs Triggered by mul-miR3954-Mediated Cleavage of *MuLnc1*

**FIGURE 3:**
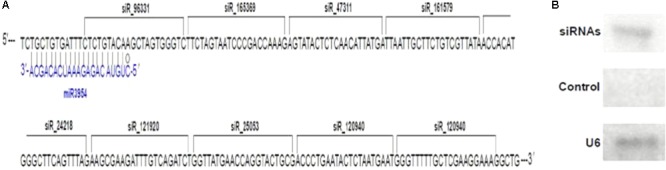
Production of secondary siRNAs from *MuLnc1* transcript. **(A)** The siRNAs matching to the *MuLnc1* transcript downstream of mul-miR3954 binding site. **(B)** Confirmation of the secondary siRNAs generated from *MuLnc1* using northern blot analysis. sRNAs extracted were used for northern blot analysis. RNA oligonucleotides complementary to the specific sequence of *MuLnc1* where the siRNAs were generated were used as probes, and RNA oligonucleotides complementary to the specific sequence of the 5′end of *MuLnc1* where no siRNA generated were used as control probes.

**FIGURE 4:**
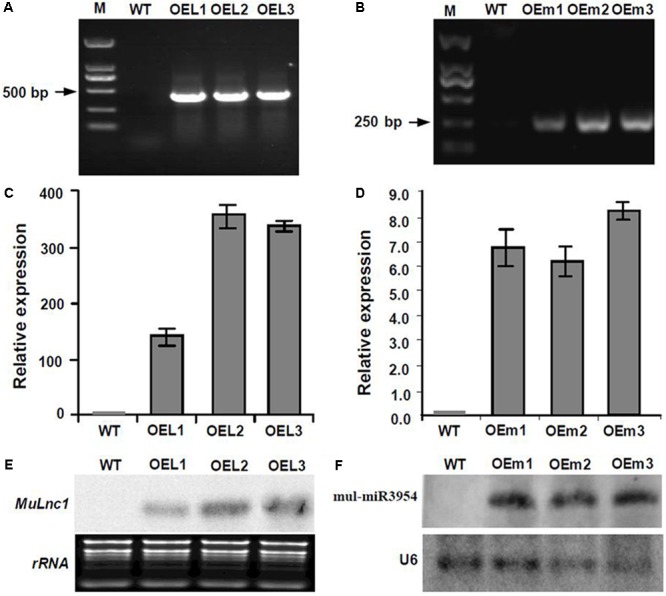
Analysis of the expression of *Mulnc1* and mul-miR3954 in the *MuLnc1* and *MUL-MIR3954* transgenic plants. **(A)** PCR analysis showing that the *MuLnc1* gene was integrated into the Arabidopsis genome **(B)** PCR analysis showing that the *MUL-MIR3954* gene was integrated into the Arabidopsis genome. **(C)** Analysis of the expressions of *MuLnc1* by qRT-PCR. The actin gene was amplified as a reference gene. **(D)** Analysis of the expressions of mul-miR3954 by qRT-PCR. The U6 gene was amplified as a reference gene. The relative gene expressions were evaluated using comparative Ct method. Assays were performed three times, each time with three replicates. Values are given as the mean ± SD of three experiments in each group. **(E,F)** Analysis of the expressions of *MuLnc1* **(E)** and mul-miR3954 **(F)** by northern blot. WT: wild type Arabidopsis. OEL1-3: *MuLnc1* transgenic lines. OEm1-3: *MUL-MIR3954* transgenic lines.

**FIGURE 5:**
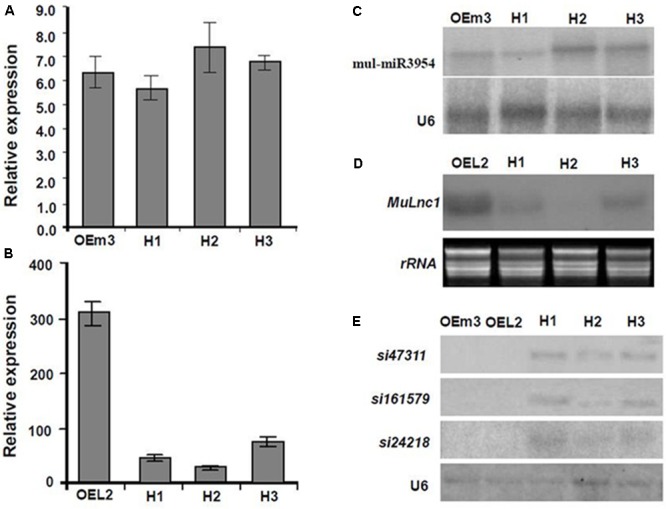
Analysis of the expression of *Mulnc1*, mul-miR3954, si47311, si161579, and si24218 in the *MuLnc1* and *MUL-MIR3954* transgenic plants and their hybrid plants. **(A,B)** Analysis of the expressions of mul-miR3954 **(A)** and *MuLnc1* **(B)** by qRT-PCR. **(C–E)** Northern blot analysis of the expressions of mul-miR3954 **(C)**, *MuLnc1* **(D)**, si47311, si161579, and si24218 **(E)**. The relative gene expressions were evaluated using comparative Ct method, and the U6 and actin genes were amplified as reference genes for mul-miR3954 and *MuLnc1* normalization, respectively. Assays were performed three times, each time with three replicates. Values are given as the mean ± SD of three experiments in each group. OEm3, the *MUL-MIR3954* transgenic line. OEL2, the *MuLnc1* transgenic line. H1-3, hybrid plants of OEL2 and OEm3 transgenic plants.

Interestingly, some siRNAs in our small RNA library sequencing data were found to match to the *MuLnc1* transcript downstream of mul-miR3954 binding site (**Figure 3A**), and northern blot results confirmed the existence of these siRNAs (**Figure 3B**). Thus, *MuLnc1* transcript might be cleaved by mul-miR3954 and generate the secondary siRNAs. Since the efficient genetic transformation system has not been established in mulberry trees, and the *MuLnc1* and mul-miR3954 do not exist in Arabidopsis, to further explore whether the *MuLnc1* is targeted by mul-miR3954, the *MuLnc1* and *MUL-MIR3954* genes were cloned and introduced into Arabidopsis plants, respectively. Genome PCR analysis results showed that both the *MuLnc1* and *MUL-MIR3954* genes were integrated into the Arabidopsis genome (**Figures 4A,B**), and qRT-PCR and northern blot analysis results indicated that the *MuLnc1* transcript and mature mul-miR3954 were successfully expressed in the transgenic Arabidopsis (**Figures 4C–F**). The hybrids between *MuLnc1* and *MUL-MIR3954* transgenic Arabidopsis were produced and used to analyze the cleavage of *MuLnc1* transcript by mul-miR3954. qRT-PCR and northern blot assays showed that there was no difference in the expression levels of mul-miR3954 between the hybrid and *MUL-MIR3954* transgenic plants (**Figure 5A**). However, the expression levels of *MuLnc1* were significantly lower in the hybrid plants than those in the *MuLnc1* transgenic plants (**Figure 5B**). This suggested that mul-miR3954 can target and cleave *MuLnc1* transcript in the hybrid plants. Moreover, four siRNAs, si47311, si161579 and si24218, generated from *MuLnc1* transcript were detected in the hybrid plants, but not detected in the *MuLnc1* and *MUL-MIR3954* transgenic plants (**Figures 5C–E**). Therefore, mul-miR3954 can cleave *MuLnc1* transcript and trigger the production of secondary siRNAs from *MuLnc1* transcript *in vivo*. Moreover, we found that these siRNAs generated from the downstream of the mul-miR3954 binding sites were in a 21-nt “phased” pattern which is a characteristic feature of ta-siRNA. Thus, these siRNAs were potential ta-siRNAs.

### Silencing of *MuCML27* Gene Guided by si161579

**FIGURE 6:**
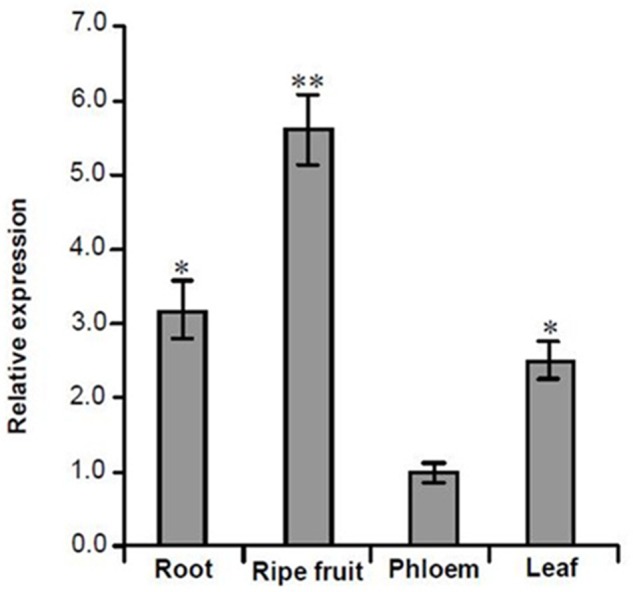
Quantitative analysis the expression levels of *MuCML27* in different tissues of mulberry using qRT-PCR. The relative gene expression was evaluated using comparative Ct method taking actin as the reference gene. Assays were performed three times, each time with three replicates. Values are given as the mean ± SD of three experiments in each group. Asterisks indicate significant difference based on Student’s *t*-test (^∗^*P* < 0.05, ^∗∗^*P* < 0.01).

**FIGURE 7:**
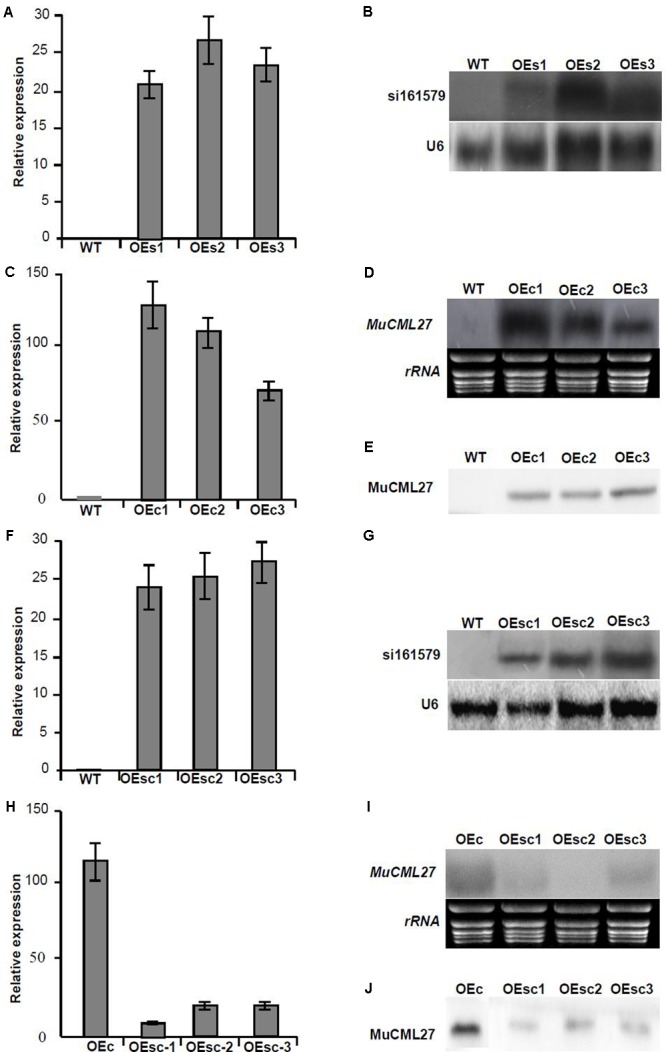
Analysis of the expression of si161579 and *MuCML27*. **(A,B)** Analysis of the expressions of si161579 in the OEs plants by qRT-PCR **(A)** and northern blot **(B)**. **(C–E)** Analysis of the expressions of *MuCML27* in the OEc plants by qRT-PCR **(C)**, northern blot **(D)** and western blot **(E)**. **(F,G)** Analysis of the expressions of si161579 in OEsc plants by qRT-PCR **(F)** and northern blot **(G)**. **(H–J)** Analysis of the expressions of *MuCML27* in the OEsc plants by qRT-PCR **(H)**, northern blot **(I)** and western blot **(J)**. The relative gene expressions were evaluated using comparative Ct method, and the U6 and actin genes were amplified as reference genes for si161579 and mRNA normalization, respectively. Assays were performed three times, each time with three replicates. Values are given as the mean ± SD of three experiments in each group. WT: wild type Arabidopsis. OEs1-3, si161579 transgenic lines. OEc1-3, *MuCML27* transgenic lines. OEsc1-3, Co-expression *MuCML27* and si161579 transgenic lines.

Analysis of the mRNA degradome data indicated that the siRNA, si161579, generated from *MuLnc1* transcript can target the mulberry calmodulin-like protein 27 gene (*MuCML27*) (**Supplementary Figure S1**). To examine whether the si161579 is functional *in vivo*, the expression level of *MuCML27* in different tissues of mulberry was determined. The results showed that the expression level of *MuCML27* was relatively high in the ripe fruits, but it was low in the roots, phloem and leaves (**Figure 6**). This suggested that there was a negative correlation between *MuCML27* and siRNA-triggering mul-miR3954, and the si161579 generated from *MuLnc1* transcript is functional and able to down regulate its target. To further verify the cleavage of *MuCML27* by si161579, the artificial vector OE-si161579 over-expressing si161579 was constructed first and then was transformed into *Arabidopsis thaliana*. Analyses of qRT-PCR and northern blot showed that the si161579 can be expressed in the transgenic *Arabidopsis thaliana* plants (OEs) successfully (**Figures 7A,B**). Moreover, the *MuCML27* gene was also cloned and transformed into *Arabidopsis thaliana*. qRT-PCR, northern blot and western blot analyses showed that the transgenic plants (OEc) expressed the *MuCML27* gene successfully (**Figures 7C–E**). Then the OEsiR-ARF vector constructed was transformed into the OEc plants, and the transgenic lines (OEsc) obtained were used to explore the si161579–mediated *MuCML27* gene silencing. qRT-PCR and northern blot analyses showed that the si161579 was successfully expressed in the OEsc transgenic Arabidopsis plants (**Figures 7F–G**). Interestingly, the mRNA and protein abundances of *MuCML27* in the OEsc plants were significant lower than those in the OEc plants (**Figures 7H–J**). These results indicated that the si161579 can silence and down-regulated the expression of *MuCML27* gene in the OEsc plants, and the silencing may occur at the level of transcription.

### Silencing of *MuCML27* Guided by si161579 Involved in Plant Response to Biotic Stress

**FIGURE 8:**
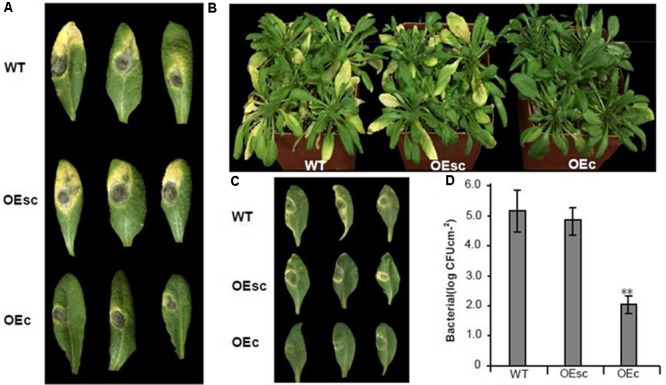
Resistance test in Arabidopsis against *B. cinerea* and *Pst* DC3000. **(A)** Symptoms observed 4 DAI on the leaves of 4-week-old Arabidopsis inoculated with 5-mm-diameter plugs of *B. cinerea*. **(B)** Disease symptoms in Arabidopsis plants sprayed with 10^6^ CFU mL^-1^ suspension of *Pst* DC3000. **(C)** Detached leaves of 4-week-old Arabidopsis plants were infiltrated with 10^6^ CFU mL^-1^ suspension of *Pst* DC 3000. Photographs were taken 3 DAI. **(D)** Bacterial populations in the inoculated leaves 36 h after inoculation with *Pst* DC3000. Assays were performed three times, each time with three replicates. Values are given as the mean ± SD of three experiments in each group. Double stars above the columns indicate significant differences according to Student’s *t*-test (*P* < 0.05). WT: wild type Arabidopsis. OEs, si161579 transgenic plants. OEc, *MuCML27* transgenic plants. OEsc, Co-expression *MuCML27* and si161579 transgenic plants.

Recent evidences suggest that several members of calmodulin-like protein (CMLs) play important roles in both abiotic and biotic responses including plant-microbe interactions (Perochon et al., 2011; Zeng et al., 2015). To find out whether the *MuCML27* gene is involved in plant responses to pathogens, the OEc and OEsc plants were inoculated with *B. cinerea* and *Pst* DC3000, respectively. Rosette leaves were detached from 4-week-old Arabidopsis plants and inoculated with *B. Cinerea*, and development of disease symptoms was analyzed 4 days after inoculation (DAI). The results showed that expanding disease yellow lesions were yielded in the leaves of wild type and OEsc plants, and the symptoms in the leaves of wild type plants were the same as those in the OEsc plants. However, the disease spot size in the inoculated leaves of OEc plants was smaller than that in the inoculated leaves of wild type and OEsc plant (**Figure 8A**). When the plants were inoculated with *Pst* DC3000, there were severe disease symptoms showing gray-brown lesion with chlorosis in the wild type and OEsc Arabidopsis plants. However, the disease symptoms were not evident in the leaves of OEc Arabidopsis plants (**Figures 8B,C**), although mild chlorosis or necrosis was occasionally observed. To determine whether the depressive symptom development reflected the restricted growth of *Pst* DC3000 inside the leaves, the bacterial growth in the inoculated leaves was monitored. Arabidopsis leaf samples were taken at 36 h after inoculation, and the strain *Pst* DC3000 reaches the concentrations of 10^5^ colony-forming units (CFU) cm^-2^ of leaf in the inoculated leaves of wild type Arabidopsis. Growth of the strain in the inoculated leaves of OEsc plants was essentially the same as that in the inoculated leaves of wild type Arabidopsis. Whereas growth of *Pst* DC3000 strains was extremely inhibited in the inoculated leaves of OEc plants (**Figure 8D**). Therefore, these results indicated that overexpressing *MuCML27* can also enhance plant resistance both to *Pst* DC3000 and *B. cinerea*, and si161579 indeed silences the *MuCML27* gene and inhibits the functions of *MuCML27* in plant response to biotic stress.

### Silencing of *MuCML27* Guided by si161579 Involved in Plant Abiotic Stress Response

**FIGURE 9:**
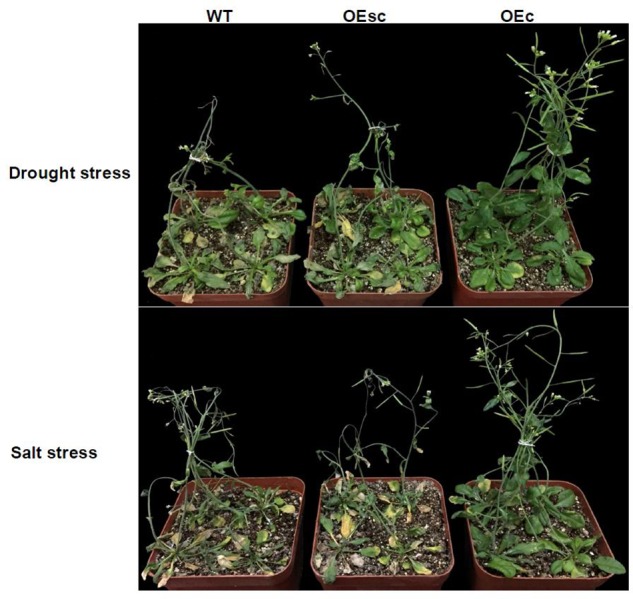
Silencing of *MuCML27* guided by si161579 involved in plant response to abiotic stress. Drought stress was imposed on 4-week-old wild type, OEsc, and OEc Arabidopsis plants grown in soil and watered with 60 gL^-1^ PEG-6000. The photographs were taken 12 d after the drought treatments showing the differences in the reactions of the plants to the drought stress. Salt stress was imposed on 4-week-old wild type, OEsc, and OEc Arabidopsis plants by irrigating with 1/8 concentrated MS solution supplemented with 250 mM NaCl. Photographs of representative seedlings were taken 2 weeks after stress. WT: wild type Arabidopsis. OEs, si161579 transgenic plants. OEc, *MuCML27* transgenic plants. OEsc, Co-expression *MuCML27* and si161579 transgenic plants.

**FIGURE 10:**
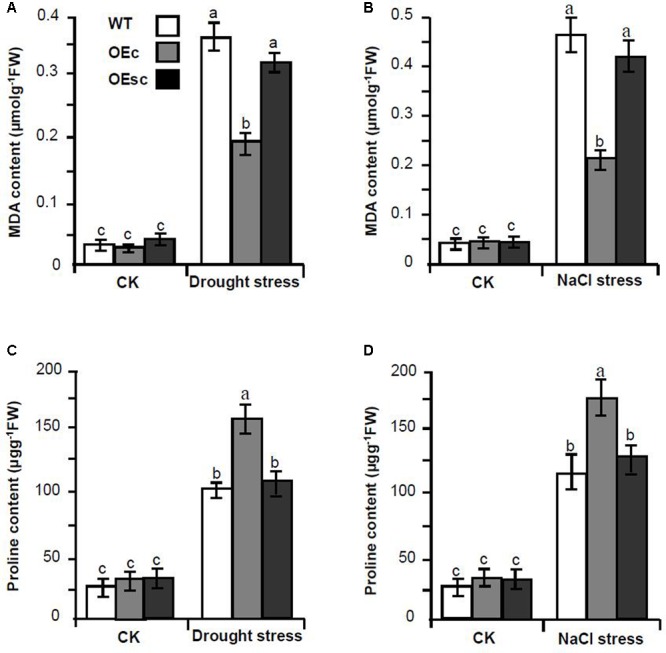
Comparison of MDA levels and proline amount between wild type and transgenic Arabidopsis plants under salt or drought stress treatments. **(A,B)** Comparison of MDA levels between WT, OEc and OEsc plants under drought **(A)** and salt **(B)** stress treatments. **(C,D)** Comparison of proline amount between OEc, OEsc, and WT plants under drought **(C)** and salt **(D)** stress treatments. Drought stress was imposed on 4-week-old wild type and transgenic Arabidopsis plants growing in vermiculite watered with 60 gL^-1^ PEG-6000. The figures were taken 12 d after the drought treatments. Salt stress was imposed on 4-week-old wild type and transgenic Arabidopsis plants by irrigating with 1/8 concentrated MS solution supplemented with 250 mM NaCl. The figures were taken two weeks after stress. CK, plant grown under normal conditions without salt or drought stress treatments. Assays were performed three times, each time with three replicates. Values are given as the mean ± SD of three experiments in each group. Different letters above the columns indicate significant differences according to Student’s *t*-test (*P* < 0.05). WT: wild type Arabidopsis. OEs, si161579 transgenic plants. OEc, *MuCML27* transgenic plants. OEsc, Co-expression *MuCML27* and si161579 transgenic plants.

To explore whether silencing of *MuCML27* mediated by si161579 is associated with plant tolerance to abiotic stress, we compared the drought and salt tolerance of the OEc, OEsc and wild-type Arabidopsis. When the plants were exposed to drought stress, the leaves of wild-type and OEsc plants became severe rolling and wilting at 12 days after withholding water. However, the leaves of OEc plants showed less rolling and wilting symptoms as compared with the wild-type and OEsc ones during the drought stress process (**Figure 9**). When the plants were exposed to salt stress, the leaves of wild-type and OEsc plants became wilted and curled after treatment with 250 mM NaCl for 2 weeks and the plants had a severe retardation in their growth. Whereas the growth inhibition level in the OEc plants was relatively less (**Figure 9**). In addition, the proline and malondialdehyde (MDA) contents in the leaves of wild type and OEc, OEsc plants under salt and drought stresses were also measured. The results showed that under salt and drought treatments, the proline level was higher but the MDA content was significantly lower in the OEc plants compared to that in the OEsc and wild type plants (**Figure 10**). These demonstrated that *MuCML27* transgenic plants exhibit increased both drought and salt tolerance, and silencing of *MuCML27* guided by si161579 inhibited the functions of *MuCML27* in plant response to drought and salt stresses.

## Discussion

Although recent studies have discovered thousands of lncRNAs, only a small number of lncRNAs have been sufficiently described, and the functions and molecular mechanisms of most lncRNAs still remain elusive (Franco-Zorrilla et al., 2007; Swiezewski et al., 2009; Heo and Sung, 2011; Ding et al., 2012; Wang et al., 2014; Wang T. et al., 2015; Chen et al., 2016). In this study, a novel lncRNA, *MuLnc1*, was identified in mulberry. Blast searches of *MuLnc1* against the Long Non-coding RNA Database v2.0 yielded none homolog, indicating it was lack of evolutionary conservation.

Plant lncRNAs may bind to specific miRNAs and function as ceRNAs (known as “target mimicry”) to protect the miRNA targets (Wang J. et al., 2015). In this study, *MuLnc1* was predicted to be one of the targets of mul-miR3954, and the negative correlation expression pattern between *MuLnc1* and mul-miR3954 and the degradome sequencing results supported the predicted results. It was reported that miR3954 was also involved in cotton responses to salt and drought stresses and was associated with drought response in tomato (Yang et al., 2013; Candar-Cakir et al., 2016). Therefore, *MuLnc1*-mul-miR3954 crosstalk might be an important regulator of gene expression in the response to environmental stress and many other biological processes in mulberry.

**FIGURE 11:**
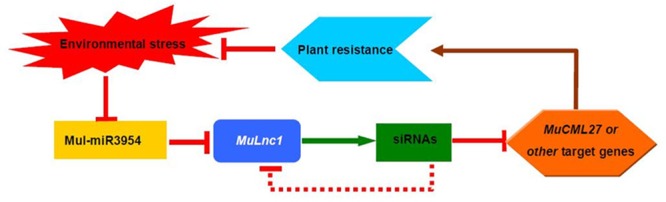
Proposed model for the complex regulatory network of mul-miR3954-*MuLnc1*-siRNA and mRNAs.

Previous studies showed that lncRNAs may function as *cis*- and trans-regulators of gene activity in many different ways (Hirsch et al., 2006; Liu et al., 2015), and some lncRNAs are processed to shorter ncRNAs such as miRNAs and siRNAs for functioning (Reinhart et al., 2002; Ben Amor et al., 2009; Jalali et al., 2012; Ma et al., 2014). Analysis of the sRNAs libraries of mulberry resulted in the identification of numerous 21-nt siRNAs that were mapped to both strands of the *MuLnc1* downstream of mul-miR3954 binding site (**Figure 3A**), suggesting that *MuLnc1* may function as siRNA precursors. It was reported that 22-nucleotide miRNAs are sufficient to trigger secondary siRNA biogenesis in plants (Chen et al., 2010; Cuperus et al., 2010). Further research confirmed that the asymmetrically positioned bulged bases in the miRNA : miRNA^∗^ duplex, regardless of miRNA or miRNA^∗^ length, determine the production of plant secondary siRNAs and are sufficient for the initiation of transitivity (Manavella et al., 2012; Liang et al., 2013). There were asymmetrically positioned bulged bases in the miRNA: miRNA^∗^ duplex of mul-miR3954 (**Supplementary Figure S2**). So mul-miR3954 may trigger the production of secondary siRNAs which were in a 21-nt “phased” pattern and were potential ta-siRNAs (**Figure 3**). Therefore, these potential ta-siRNAs may regulate the expression of *MuLnc1* transcript in *cis*; moreover, they may function in *trans* to target other genes. Our data showed that one of the siRNAs, si161579, can target and silence the expression of the *MuCML27* gene belonging to the CML family whose members are important Ca^2+^ sensors and play significant roles in mediating plant stress tolerance (White and Broadley, 2003; Chinpongpanich et al., 2011, 2012; Vadassery et al., 2012). Our results showed that the *MuCML27* transgenic plants exhibited increased resistance to biotic and abiotic stresses. Besides the si161579, the siRNAs originated from the *MuLnc1* were predicted to target many other protein-coding genes with diverse functions. Therefore, as the precursor of these siRNAs, the *MuLnc1* gene may be involved in different physiological processes, and there was a complex regulatory network of mul-miR3954-*MuLnc1*-si161579-*MuCML27* involved in the response to environmental stress in mulberry. Based on the information obtained, the complex regulatory network of mul-miR3954-*MuLnc1*-siRNAs-*MuCML27* involved in mulberry response to environmental stress is outlined in **Figure 11**.

In addition, it was reported that lncRNAs are widely involved in regulation of gene expression including transcription, mRNA splicing and translation, and can affect the stability of RNA-protein complexes and regulate the activities of proteins (Zhu and Wang, 2012; Kornienko et al., 2013; Chekanova, 2015). Since the complete genome information of mulberry is not available, this hinders predicting the target genes and the interaction protein of *MuLnc1*. So more research is needed to understand the functions of *MuLnc1*, and *MuLnc1* can be used as a target for genetic improvement of mulberry in the future.

## Conclusion

The novel lncRNA, *MuLnc1*, was found to be cleaved by mul-miR3954 and produce secondary siRNAs in a 21-nt “phased” pattern in mulberry. One of the siRNAs produced, si161579, can silence the expression of the *MuCML27* gene, and the transgenic Arabidopsis plants ectopically expressing *MuCML27* gene showed more resistant to biotic and abiotic stresses. Therefore, there was a complex regulatory network of mul-miR3954-*MuLnc1*-si161579-*MuCML27* involved in mulberry response to environmental stresses. All of these, taken together, suggest that *MuLnc1* is associated with environmental stress in mulberry, and has potential applications in mulberry genetic improvement in the future.

## Notes

Raw data of transcriptome, sRNA datasets and degradome can be obtained from SRA database with the accession number SRP139984, SRP139984, and SRP140041, respectively.

## Author Contributions

Y-PG and X-LJ designed the research. S-SY generated and characterized Arabidopsis transgenics. Y-NZ performed quantitative RT-PCR. H-NZ and H-LZ designed and performed northern and western blot assays. X-LJ wrote the manuscript with input from all authors. All authors read and approved the final manuscript.

## Conflict of Interest Statement

The authors declare that the research was conducted in the absence of any commercial or financial relationships that could be construed as a potential conflict of interest.

## Footnotes

1 http://plantgrn.noble.org/psRNATarget/

2 http://cpc.cbi.pku.edu.cn

## Supplementary Material

FIGURE S1 Identification of *MuCML27* as a target of si161579 in mulberry by degradome sequencing.

FIGURE S2 Predicted secondary structure of mul-miR3954. Red indicates miRNA guide strand and black arrows mark asymmetric bulges.

TABLE S1 The sequences of northern blot probes.

TABLE S2 The primers used for qRT-PCR.

TABLE S3 The primers used for gene cloning and vector construction.

TABLE S4 Sequences of *Mul-MIR3954, MuLnc1*, and *MuCML27* genes.
