# Supplementary figures and images for: A Novel LncRNA, MuLnc1, Associated With Environmental Stress in Mulberry (Morus multicaulis)
Source: Front Plant Sci. 2018 May 29;9:669. doi: 10.3389/fpls.2018.00669 (PMC5987159; doi:10.3389/fpls.2018.00669)

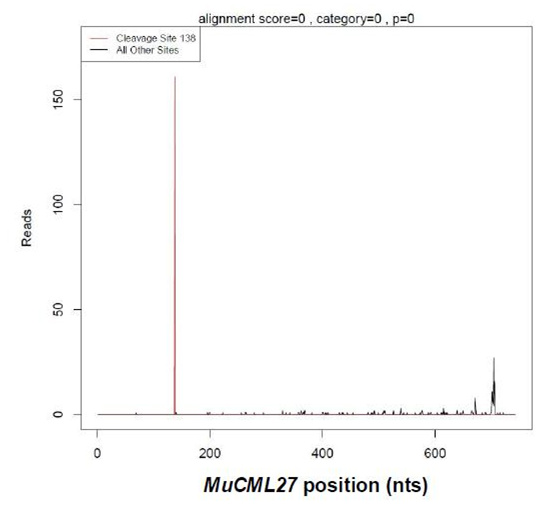

Supplement: FIGURE S1 — Identification of MuCML27 as a target of si161579 in mulberry by degradome sequencing. [file Image_1.JPEG]

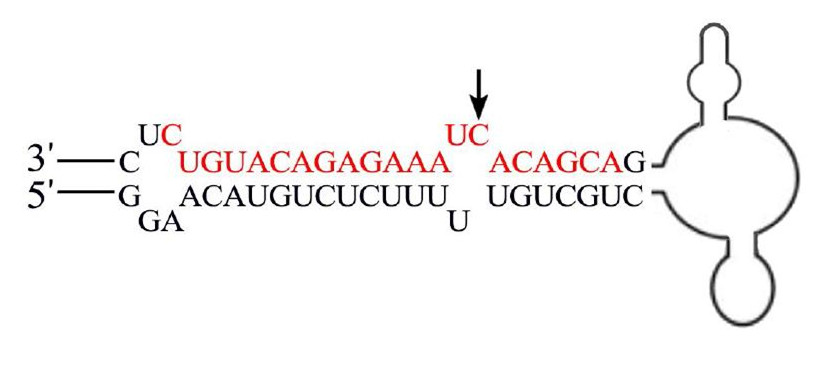

Supplement: FIGURE S2 — Predicted secondary structure of mul-miR3954. Red indicates miRNA guide strand and black arrows mark asymmetric bulges. [file Image_2.JPEG]
