# Supplementary material for: A Novel LncRNA, MuLnc1, Associated With Environmental Stress in Mulberry (Morus multicaulis)
Source: Front Plant Sci. 2018 May 29;9:669. doi: 10.3389/fpls.2018.00669 (PMC5987159; doi:10.3389/fpls.2018.00669)
Supplement: TABLE S1 — The sequences of northern blot probes. [file Table_1.DOC]

**Table S1. The primers used for** **qRT-PCR.**

| **Primer names** | **Sequence (5'→3')** |
| --- | --- |
| mul-miR3954 | CTGTACAGAGAAATCACAGCA |
| *mul-U6* | *ATGGCCCCTGCGTAAGGATGA* |
| *ath-U6* | *TGGCCCCTGCGCAAGGATGA* |
| *MuCML27* F | TTCGATCTCTACGATGTGGAC |
| *MuCML27* R | GCGACGGTGTTAGTCATCATC |
| *MuLnc1* F | GCTAATGGGTCTTCTAGTAATC |
| *MuLnc1* R | ATTCCTCTGCCTCATCAGCCT |
| *mul-actin F* | CACTGAGGCTCCTTTGAACCC |
| *mul-actin R* | AGGTCGAGACGGAGAATAGCATG |
| *ath-actin F* | *GCACCCTGTTCTTCTTACCG* |
| *ath-actin R* | *AACCCTCGTAGATTGGCACA* |
