# Supplementary material for: A Novel LncRNA, MuLnc1, Associated With Environmental Stress in Mulberry (Morus multicaulis)
Source: Front Plant Sci. 2018 May 29;9:669. doi: 10.3389/fpls.2018.00669 (PMC5987159; doi:10.3389/fpls.2018.00669)
Supplement: TABLE S2 — The primers used for qRT-PCR. [file Table_2.DOC]

**Table S2. Sequences for northern blot probes.**

| **Probe name** | **Probe sequence (5'→3')** |
| --- | --- |
| *MuLnc1* siRNA-producing region | CUUUCCUUCGAGCUUUUUCCCAUUCAUUAGAGUAUUCAGGGUCGCAGUACCUGGUUCAUAACCAGAUCUGACAAAUCUUCGCUUCUAAACUGAAGCCCAUGUGGUUAUAACGACAGAAGCAAUUAAUCAUAAUGUUGAGAGUAUACUCUUUGGUCGGGAUUACUAGAAGACCCAUUAGCUUGUACAGAG |
| 5'end of *MuLnc1* where no siRNA generated | CAUUUCCACAAUUUUGCCCAACAAUUGAUUGAAACGAACGACACAAUGCAGAUGACGCAUUUGGAUAAUUUCAUCAAACAAAUACAAGGCAUCUUUAAGAGUUGUUAUGUCAAUGCUUCUCCCAUGGUUUUCGUCAUUUGG |
| *MuCML27* | GAAUCGAGAUCCUCUAUCACGCGCUGGAGAUCCUUGGGCAUAACCGUUAUGCCUUGGGCUUUGAGCGCCUCGCCUAGCUCCGCGACUGAGAUCUUGCCGUCGCCGUUGGCGUCGAAUUGAUUGAACACCUUCUCGAUCUCGUCGGGGUUCUGGAGGUACUGTAACGGCUUCGAUUUGUUGGAUUCGGUGUUAAUUGCUUCUGUCGUCAU |
| mul-miR3954 | UGCUGUGAUUUCUCUGUACAG |
| si165369 | CUUUGGUCGGGAUUACUAGAA |
| si47311 | UCAUAAUGUUGAGAGUAUACU |
| si161579 | UAUAACGACAGAAGCAAUUAA |
| si24218 | CUAAACUGAAGCCCAUGUGGU |
