# Supplementary material for: A Novel LncRNA, MuLnc1, Associated With Environmental Stress in Mulberry (Morus multicaulis)
Source: Front Plant Sci. 2018 May 29;9:669. doi: 10.3389/fpls.2018.00669 (PMC5987159; doi:10.3389/fpls.2018.00669)
Supplement: TABLE S3 — The primers used for gene cloning and vector construction. [file Table_3.DOC]

**Table S3. The primers used for** **gene cloning and vector construction.**

| **Primer names** | **Sequence (5'→3')** |
| --- | --- |
| miR165 F | ATTTGTGAATCTGCTAAGATCG |
| miR165 R | CCAAGCTTTTGTAATTTGCGC |
| si161579 I | TTAATTGCTTCTGTCGTTATAATATTATAGAT |
| si161579 II | TATAACGACAGAAGCAATTAACCTCAACTGA |
| si161579 III | TATAACGACAGAAGCAATTAACCAACATGTT |
| si161579 IV | TTAATTGCTTCTGTCGTTATAAGGATACTCT |
| *Mul-MIR3954* F | ATATCCCTATCTACAGCAAAGG |
| *Mul-MIR3954* R | TAGGATACACACAAATACCAATCC |
| *MuCML27* F | ATGACGACAGAAGCAATTAAC |
| *MuCML27* R | TCAAGCATCGTTGCCGTTGCTG |
| *MuLnc1* F | CATTCTCAACCCTTTCCAAATG |
| *MuLnc1* R | CTCCTCTAGTCATTTTGCGAAAG |
