# Supplementary material for: A Novel LncRNA, MuLnc1, Associated With Environmental Stress in Mulberry (Morus multicaulis)
Source: Front Plant Sci. 2018 May 29;9:669. doi: 10.3389/fpls.2018.00669 (PMC5987159; doi:10.3389/fpls.2018.00669)
Supplement: TABLE S4 — Sequences of Mul-MIR3954, MuLnc1, and MuCML27 genes. [file Table_4.DOC]

**Table S4. Sequences of *Mul-MIR3954*, *MuLnc1*, and *MuCML27* genes.**

| Gene names | **Sequence (5'→3')** |
| --- | --- |
| Mul-MIR3954 | ATATCCCTATCTACAGCAAAGGAAAAGGGTCCAATCCATTTCTCTGTACAGAGAAATCACAGCAGCGATCAAAGAGGTTTCTCATCTCTTTCATCGAATCAAGTTTCTTTCACTCTCTGCTGTTTTTCTCTGTACAAGGAGATGTATTAGATCCGCTCAATAATATACTGTCTTTGATGAAATCAGTCATGTTTCAGTGATTTTCTGGATTGGTATTTGTGTGTATCCTACTTTACATTAAAGTAGTTTTAACAGATTTAGATTCAAAAAAAAAAAAAAAAA |
| *MuLnc1* | GCTCAAAATCATTCTCAACCCTTTCCAAATGACGAAAACCATGGGAGAAGCGTTGACATAACAACTCTTAAAGATGCCTTGTATTTGTTTGATGAAATTATCCTAATGCGTCCTCTGCCTTGTGTCGTTCGTTTCAATCAATTGTTGGGCAAAATTGTGGAAATGGAAAACTTTTCTGCTGTGATTTCTCTGTACAAGCTAGTGGGTCTTCTAGTAATCCCGACCAAAGAGTATACTCTCAACATTATGATTAATTGCTTCTGTCGTTATAACCACATGGGCTTCAGTTTAGAAGCGAAGATTTGTCAGATCTGGTTATGAACCAGGTACTGCGACCCTGAATACTCTAATGAATGGGTTTTTGCTCGAAGGAAAGGCTGATGAGGCAGAGGAATGCTTTCGCAAAATGACTAGAGGAG |
| *MuCML27* | TGCTCTCTCTCTCTCTCTCTCTCTACATCTCTATATATAAGGCATAACAGACTCTCCATTTTAATCCTTAAAATCCGAATTCCGAATTCAAAAGCAAGTAAAACTTATCTTACTCAATCGATCAACTATGACGACAGAAGCAATTAACACCGAATCCAACAAATCGAAGCCGTTACAGTACCTCCAGAACCCCGACGAGATCGAGAAGGTGTTCAATCAATTCGACGCCAACGGCGACGGCAAGATCTCAGTCGCGGAGCTAGGCGAGGCGCTCAAAGCCCAAGGCATAACGGTTATGCCCAAGGATCTCCAGCGCGTGATAGAGGATCTCGATTCCGACCGCGACGGCTTCATTTCGGTGAAGGAATTCGCCGCCTTCTGCAGCCAGGGCTCCGACGATGGCGGTGCCGCTGAGCTCCGCGACGCCTTCGATCTCTACGATGTGGACAAGAACGGCCTCATTTCCGCCGAGGAGCTCCACAAAGTCCTCAACCGCCTCGGCATGAAATGCTGCCCCGAAGACTGCCACCGCATGATCACCTCTGTCGATTCCGACGGCGACGGCAACGTCAACTTCACTGAGTTCAAAAAGATGATGACTAACACCGTCGCCAGCAACGGCAACGATGCTTGATTCGCGAGATCGACGGCGGATCGATGACGAGCGCGTCAATGTACGCGCCGTTTCCTCTTAAAGCCTTTCTAAATCTAAATCATTAGC |
